# A Case of Saphenous Vein Graft Aneurysm Treated With Percutaneous Coiling

**DOI:** 10.7759/cureus.49262

**Published:** 2023-11-22

**Authors:** Ashwin Jagadish, Shobha Hiremagalur, Ahmed Khan

**Affiliations:** 1 Internal Medicine, East Tennessee State University James H. Quillen College of Medicine, Johnson City, USA; 2 Cardiology, Ballad Health CVA Heart Institute, Johnson City, USA

**Keywords:** percutaneous coiling, coronary artery bypass grafting, saphenous vein graft aneurysm, internal medicine, cardiology

## Abstract

In adults, coronary artery bypass grafting (CABG) is a commonly performed surgery. Oftentimes, saphenous veins from the lower extremity are used as the graft vessels. A rare complication of this procedure is the formation of saphenous vein graft (SVG) aneurysms. We present the case of a 63-year-old male who presented to a referring emergency department with left-sided chest pain after falling off a ladder. The patient’s initial chest X-ray revealed a suprahilar mass that was suspicious for being an aneurysm. A computerized tomography coronary angiogram indicated a large aneurysm. The patient was transferred to our facility for specialist evaluation. The patient’s history was positive for two CABG procedures and a sternal wound infection, so a repeat sternotomy was not advisable. The SVG aneurysm was treated with percutaneous coiling. The patient tolerated the procedure well and was discharged home the next day.

## Introduction

Coronary artery bypass grafting (CABG) is the most frequently performed cardiac surgery in adults [1]. Although the United States has seen a reduction in procedural volume, more than 200,000 CABGs are performed annually [2]. A rare complication of using a saphenous vein graft (SVG) while performing CABG is the formation of an aneurysm [3]. Presentation of SVG aneurysm can range from asymptomatic to rupture or death [2]. We present the case of a patient who had a large SVG aneurysm that was ultimately treated with percutaneous coil embolization of the aneurysm.

## Case presentation

A 63-year-old male with a history of type 2 diabetes mellitus, essential hypertension, and dyslipidemia presented to the emergency room of a referring hospital due to left-sided chest pain that persisted for two days after falling off a ladder. His cardiac surgical history included multiple percutaneous coronary interventions, coronary artery bypass grafting (CABG) at the ages of 39 and 55, and debridement for sternal wound infection after CABG. Since his most recent CABG, he has not received further cardiac catheterization.

On presentation to the initial emergency department, his blood pressure was 158/98 mmHg and his heart rate was 63 beats per minute. He reported subjective chest, pain which was worsened by coughing and sneezing. On physical examination, there was mild tenderness to palpation of the anterior and left chest walls. The patient’s complete blood count and basic metabolic panel revealed results within normal limits, other than a non-fasting blood glucose level of 176 mg/dL (normal range: 70-99 mg/dL). A troponin level was not obtained at that time.

**Figure 1:**
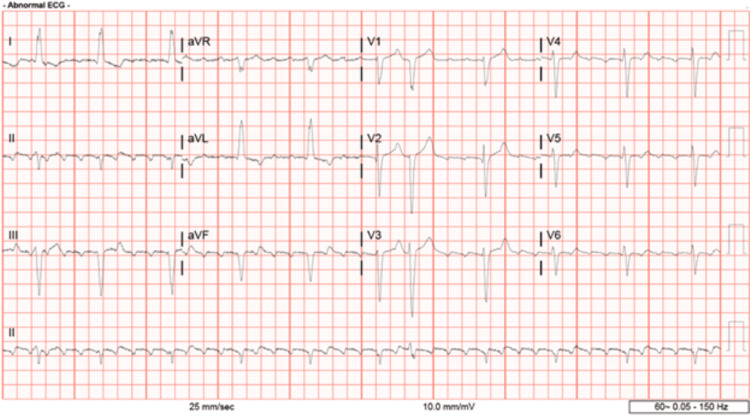
Electrocardiogram Demonstrating Atrial Flutter

**Figure 2:**
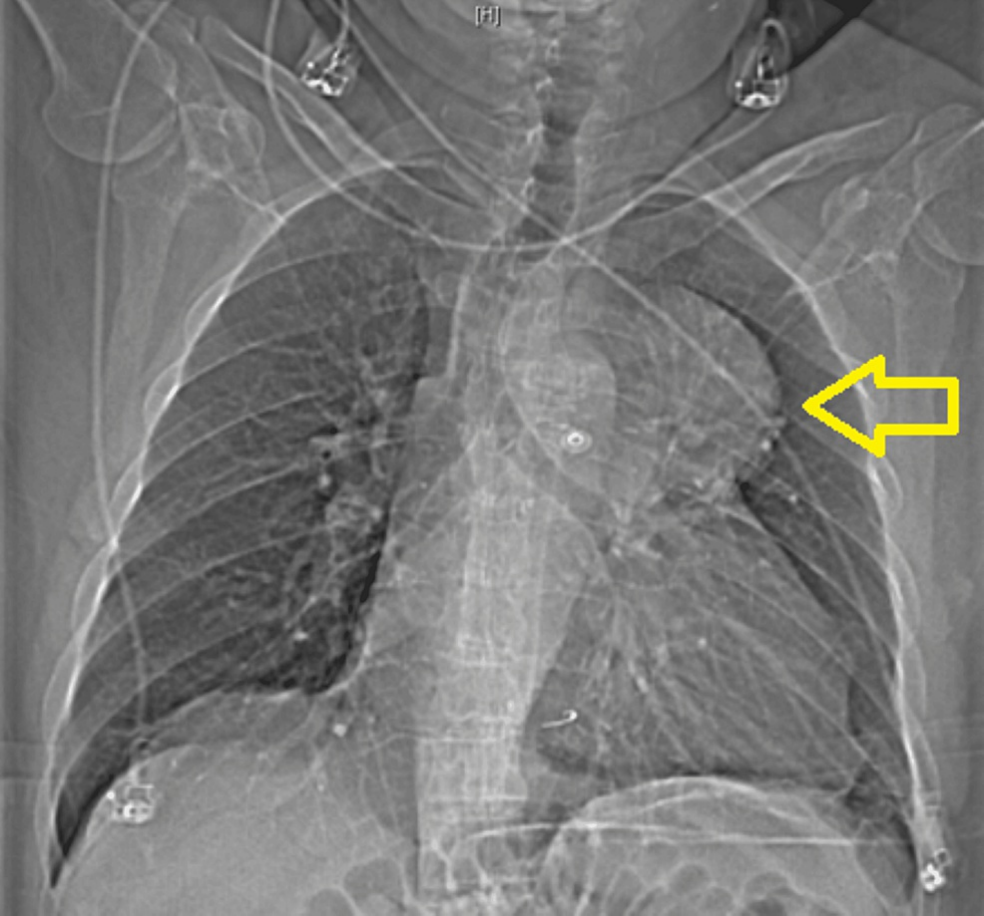
Chest X-ray Demonstrating Suprahilar Mass The arrow indicates the suprahilar mass.

**Figure 3:**
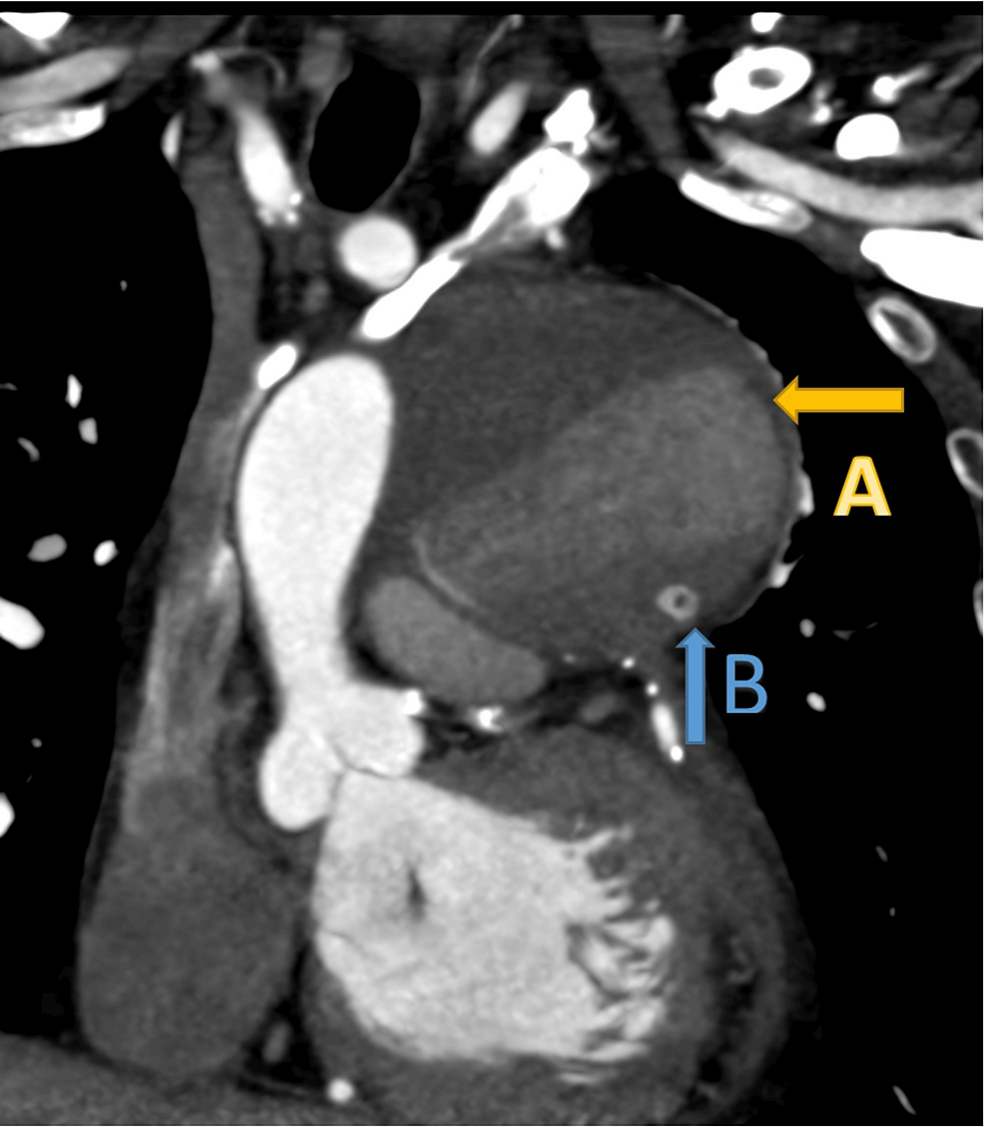
Computerized Tomography Coronary Angiogram (A) Aneurysm of the saphenous vein graft; (B) Stent within the aneurysm

An electrocardiogram was performed and revealed atrial flutter with controlled ventricular response (Figure 1). A chest X-ray revealed the presence of a suprahilar mass, possibly an aneurysm (Figure 2). As a result, a computerized tomography coronary angiogram was then obtained. The results demonstrated a large aneurysm measuring 8.6 x 7.9 x 7.4 cm, which appeared to arise from a saphenous vein graft presumably to a diagonal artery. In addition, the SVG appeared to be occluded at its distal end, and a stent appeared to be present within the aneurysm (Figure 3).

**Figure 4:**
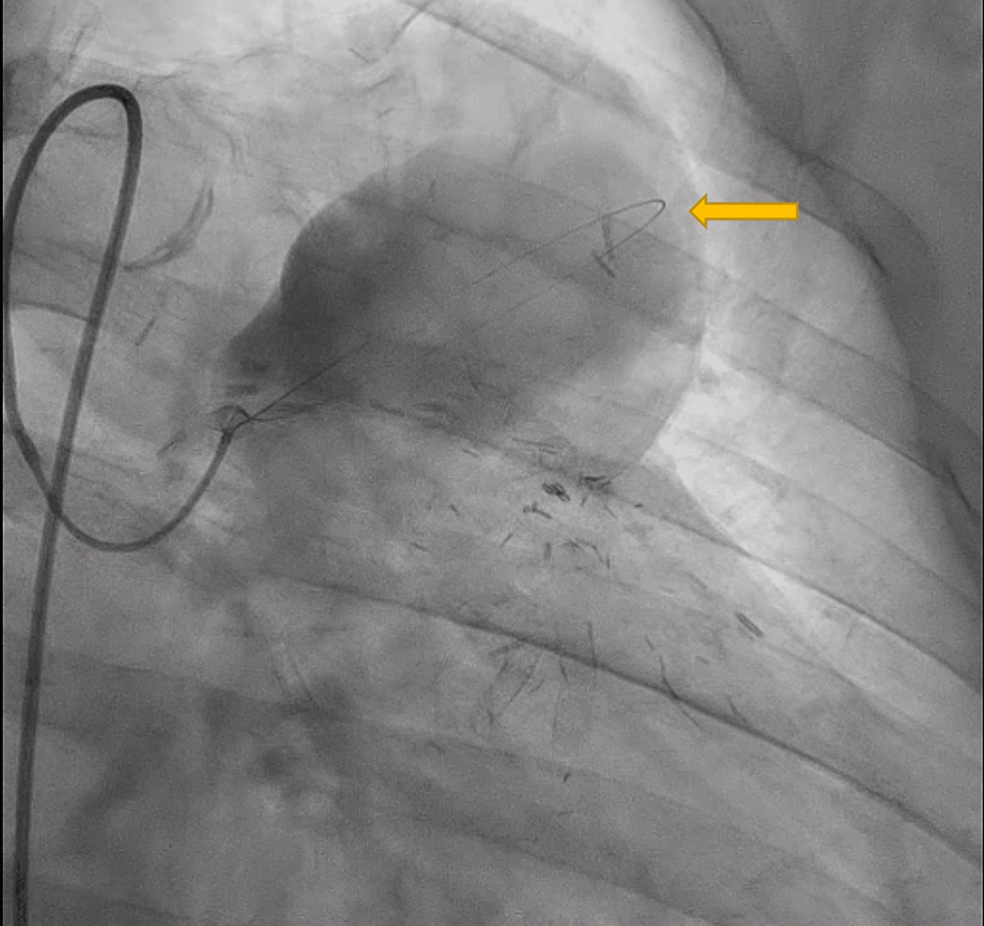
Cardiac Catheterization Demonstrating a Saphenous Vein Graft Aneurysm The arrow indicates a saphenous vein graft aneurysm.

**Figure 5:**
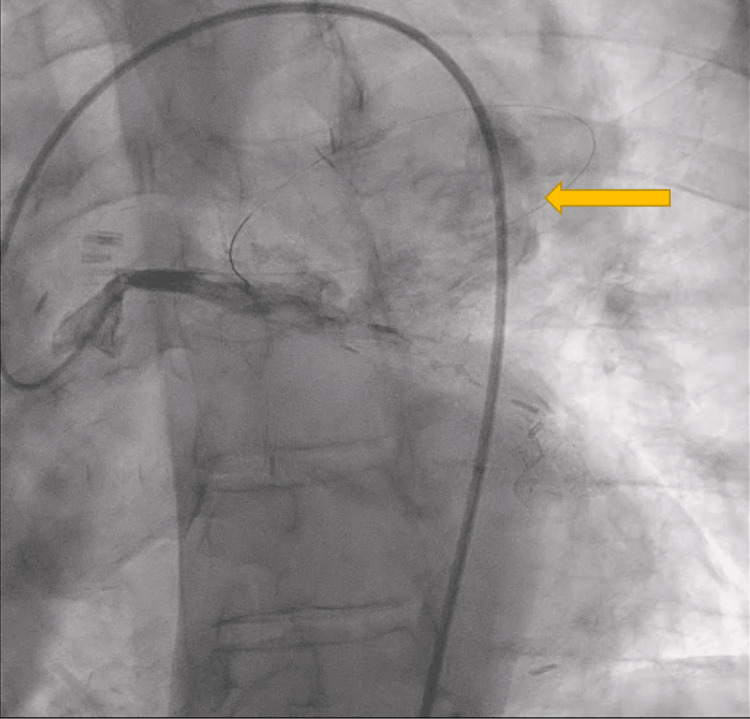
Cardiac Catheterization Demonstrating a Saphenous Vein Graft Aneurysm The arrow indicates a saphenous vein graft aneurysm.

Due to the size of the aneurysm, the patient was transferred to our facility for evaluation by the cardiothoracic (CT) surgery team. On presentation, his echocardiogram demonstrated a left ventricular ejection fraction of 35-40%. The CT surgeon recommended that the interventional cardiology team pursue cardiac catheterization. This procedure confirmed the presence of an actively filling large aneurysm arising from the proximal portion of a saphenous vein graft, presumably to a diagonal artery (Figures 4, 5). Furthermore, the vein graft appeared to be occluded at its distal end. Critical stenosis of 95% was noted in the SVG to the right posterior descending artery (PDA), and the proximal part of a large native circumflex artery was noted to have 99% stenosis. The left internal mammary artery (LIMA) graft to the left anterior descending artery (LAD) was patent as was a saphenous vein graft to one obtuse marginal branch. The native LAD and right coronary artery were completely occluded in their proximal regions. A diagonal branch was noted to be filling in a retrograde manner from the LAD when the LIMA was injected with contrast.

Due to the patient’s history of multiple CABG procedures and sternal wound infection, the CT surgeon believed that percutaneous intervention would be safer than a repeat sternotomy. Out of concern for potential aneurysm rupture, a decision was made to percutaneously close the SVG and the arising aneurysm using coils. Initially, a successful percutaneous intervention was performed on the SVG to the right posterior descending artery and the proximal part of a large native circumflex artery

**Figure 6:**
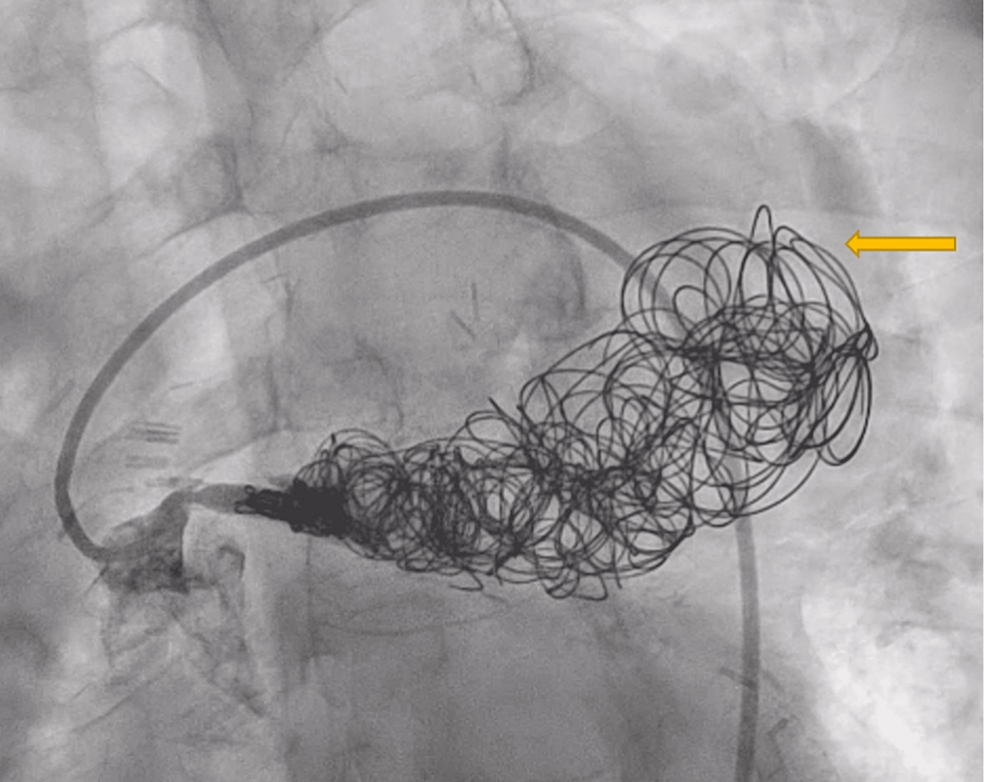
Percutaneous Coiling of the Saphenous Vein Graft and the Aneurysm The arrow indicates the coils.

For the coiling procedure, the aortocoronary SVG was engaged with a 6 French guide catheter, and selective angiography was performed using a 5.5 French Guideliner. The vein graft was wired with a Whisper wire, and a Progreat microcatheter (Terumo) was advanced into the ectatic segment of the vein graft. Afterward, a 20 mm X 50 cm Azur coil (Terumo; Tokyo, Japan) was advanced through the Progreat microcatheter and deployed into a vein graft. Additional coils were deployed to occupy the potential space. Smaller coils were deployed in the partially occluded stent in the proximal portion of the SVG to prevent flow into the ectatic graft. Final angiograms revealed the absence of flow in the ectatic graft (Figure 6). The patient tolerated the procedure well, and he was discharged home the following day.

**Figure 7:**
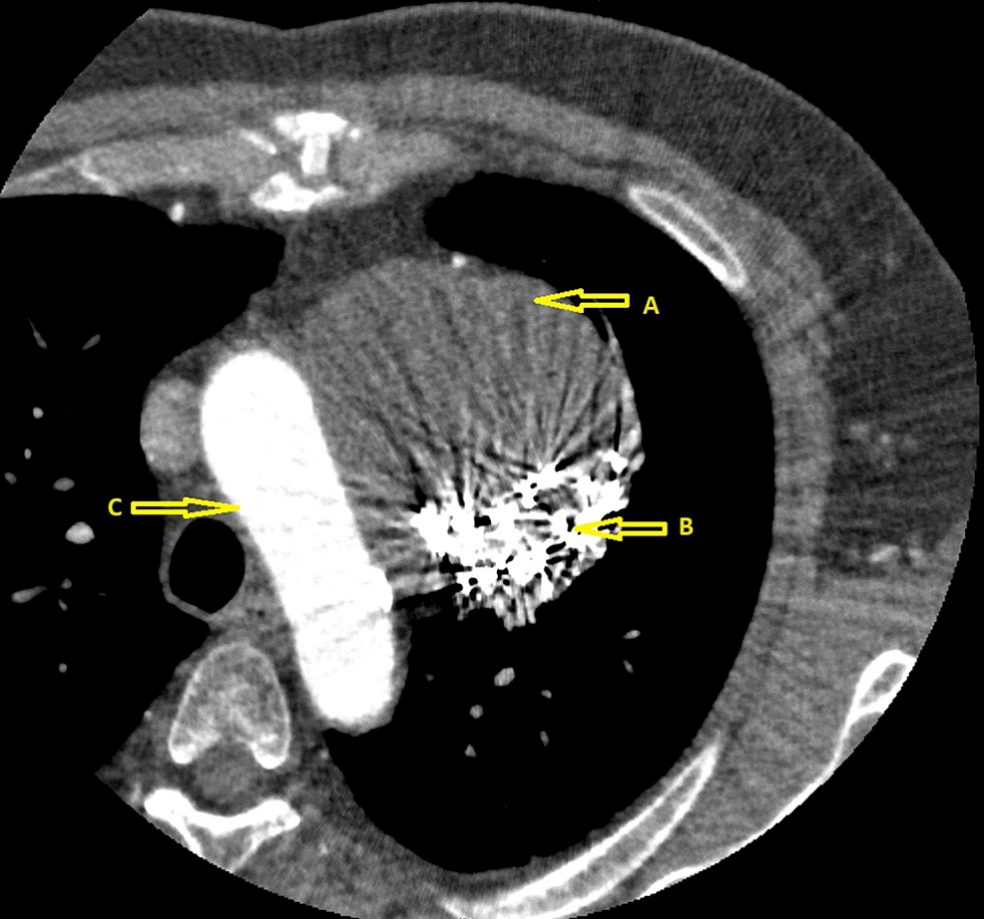
Computerized Tomography Angiography After Coiling (A) Thrombosed SVG aneurysm; (B) Coil; (C) Aorta SVG: saphenous vein graft

Three weeks later, the patient underwent successful direct current synchronized cardioversion from atrial flutter to sinus rhythm. Seventeen months later, an elective computerized tomography angiography was performed to reassess the SVG aneurysm. The imaging revealed a completely thrombosed SVG aneurysm and complete proximal occlusion of the SVG (Figure 7).

## Discussion

Coronary artery bypass grafting (CABG) is a surgical procedure performed to restore blood flow to an ischemic myocardium [4]. It involves using graft vessels to bypass obstructions in the coronary system, with the goal of re-establishing functionality and alleviating signs of angina [4]. While vessels such as the radial artery, right internal mammary artery, and gastroepiploic artery can be used as grafts, the most common grafts are the left internal mammary artery and lower extremity saphenous veins [4]. Common complications of CABG include infections of the sternum, pneumonia, venous thromboembolism, occlusion of the bypass graft, postoperative atrial fibrillation, pulmonary hypertension, pericardial effusion, cardiac tamponade, stroke, and acute kidney injury [5]. The incidence of a rare complication, SVG aneurysm, can range from 0.07% to 14% of individuals [2]; it has been seen in those who have undergone CABG one-to-two decades prior to the finding [3].

An aneurysm is described as a vessel expanding to more than 1.5 times the size of the vessel being referenced [2]. There does not appear to be any safe size to merely monitor SVG aneurysms [6]. It has been reported that 20 mm aneurysms can be correlated with a 33.3% rate of complications while aneurysms greater than 100 mm in size have a complication rate of up to 69.2% [6].

Generally, SVG aneurysms are asymptomatic and are identified while conducting imaging for other medical conditions [2]. When patients have symptoms, they may present with chest pain, shortness of breath, hemoptysis, or aneurysmal rupture resulting in death [2].

Imaging is often conducted to verify the diagnosis, size, and presence of complications of SVG aneurysms; although, there is no agreement on the best imaging modality [6]. One literature review noted that approximately two-thirds of cases involved cardiac catheterization, three-fifths involved computerized tomography, one-eighth involved magnetic resonance imaging, and slightly more than half involved chest X-rays [6]. Echocardiograms were obtained in less than 30% of cases [6].

In the event that the SVG aneurysm is complicated by compression of adjacent cardiac structures or the presence of a fistula or rupture, surgical intervention may be the only feasible option [6]. However, in other cases, percutaneous interventions are realistic alternatives [2].

## Conclusions

Patients who undergo CABG have a rare chance of developing SVG aneurysms. Many SVG aneurysms are asymptomatic and identified through imaging for other presenting symptoms. So far, a safe size for monitoring the aneurysm has not been established. Imaging is often utilized to obtain a formal diagnosis, and treatment can include surgical or percutaneous interventions.
